# Primary Biliary Cirrhosis-Specific Antimitochondrial Antibodies in Neonatal Haemochromatosis

**DOI:** 10.1155/2013/642643

**Published:** 2013-09-19

**Authors:** Daniel S. Smyk, Maria G. Mytilinaiou, Tassos Grammatikopoulos, A. S. Knisely, Giorgina Mieli-Vergani, Dimitrios P. Bogdanos, Diego Vergani

**Affiliations:** ^1^Institute of Liver Studies, GI and Nutrition Centre, King's College London School of Medicine at King's College Hospital, Denmark Hill Campus, London SE5 9RS, UK; ^2^Paediatric Liver, GI and Nutrition Centre, King's College London School of Medicine at King's College Hospital, Denmark Hill Campus, London SE5 9RS, UK; ^3^Department of Medicine, Faculty of Medicine, School of Health Sciences, University of Thessaly, 41110 Biopolis, Larissa, Greece

## Abstract

*Background and Aim*. Neonatal hemochromatosis (NH) is characterised by severe liver injury and extrahepatic siderosis sparing the reticuloendothelial system. Its aetiology is obscure, although it has been proposed as an alloimmune disease, resulting from immunological reaction to self-antigens (alloantigens) which the body recognizes as foreign. We studied an infant with NH and his mother whose sera contained antimitochondrial antibody (AMA), the hallmark of primary biliary cirrhosis (PBC). *Material and Methods*. To investigate the origin of AMA in the infant, we studied isotype distributions in serum from the mother and infant. Serum samples were obtained at diagnosis of NH, after liver transplantation (LT; age 1 month), and over the ensuing 17 months. *Results*. At NH diagnosis, infant and maternal serum contained AMA of the IgG isotype, predominantly of the G3 and G1 subclasses. AMA strongly reacted against the pyruvate dehydrogenase complex E2 subunit (PDC-E2), the major PBC-specific AMA autoantigen. Anti-PDC-E2 responses in both infant and mother declined over time, being present 2 months after LT (mother and child) and absent 10 months later (mother) and 17 months later (child). *Conclusion*. The association of maternally transferred IgG1 and IgG3 subclass AMA with the appearance of liver damage in an infant with NH may suggest a causal link between antibody and liver damage.

## 1. Introduction

Neonatal haemochromatosis (NH) is a rare condition of unknown aetiology characterised by perinatal liver failure and extrahepatic siderosis sparing the reticuloendothelial system [1, 2]. No genetic or infectious factors have been identified to explain its recurrence rate of 60–80% in pregnancies that follow an index case [1, 3–7]. The recent suggestion that NH is an alloimmune disease is based on a recurrence pattern similar to that of other alloimmune diseases, on the ability of administration of intravenous immunoglobulin during pregnancy substantially to ameliorate or even to prevent fetal liver disease in siblings of an index patient, and on the demonstration that patient hepatocytes bear assembled components of the terminal complement cascade pathway together with IgG of maternal origin [2, 7–11].

A direct proof that NH is alloimmune is missing since no target has been identified for the putative alloantibody [9, 12]. Autoantibodies of maternal origin, such as anti-Ro and anti-La, have been described in some children with NH [3, 13–17]. The relevance of these antibodies, which are not liver specific [18], to the pathogenic process in NH is unclear, although they have been associated with liver disease in rare cases [19]. 

We investigated an infant with NH whose serum contained antimitochondrial antibody (AMA), the hallmark of primary biliary cirrhosis (PBC), a disease with a striking (>95%) female preponderance typically affecting middle-aged women [20, 21]. We describe the characteristics of the AMA found in this infant, demonstrate its maternal origin, and discuss its potential relevance to the development of NH.

## 2. Material and Methods

### 2.1. Subjects

Serum samples from a mother and her infant, who had NH, were obtained. This was the first pregnancy of a 27-year-old of Eastern European origin with no significant medical history complicated by oligohydramnios, symmetrical intrauterine growth retardation, and reduced fetal movements. After spontaneous labour at term she was delivered vaginally of a boy weighing 2.270 kg (small for gestational age; < second centile) with a head circumference of 33 cm (<0.4 centile). Apgar scores were not recorded and the placenta was not examined; the infant was described as in good condition. Left hip dislocation was noted.

Evaluation for poor feeding and hypoglycaemia at age 2 days identified jaundice with coagulopathy [INR 2.3, normal value (nv) <1.2], resistant to parenteral vitamin K. Liver synthetic function was poor [serum albumin 22 g/L, normal range (nr): 35–50]. Indices of hepatic and renal function declined despite treatment for presumed sepsis and the boy was transferred to the Paediatric Liver Centre at King's College Hospital at 7 days of age. NH was suspected in view of the patient's age, history, and clinical-laboratory test results [serum ferritin 1481 ng/mL (nr: 20–300), INR 5.37, albumin 26 g/L, AST 44 IU/L (nr: 10–50), ALT 30 IU/L (nr: 10–50), and total bilirubin 292 *μ*mol/L (nr: 3–20)]. Before blood transfusion, total iron binding capacity was 28 *μ*mol/l (nr: 50–72) and transferrin saturation was 72% (nr: 20–50%) in peripheral-blood serum. Serum immunoglobulin concentrations were IgG, 5.53 g/L (nr: 5–13); IgM, 0.63 g/L (nr: 0.05–0.2); and IgA, 0.63 g/L (nr: 0.01–0.08). Autoantibody testing by indirect immunofluorescence and molecular assays revealed the presence of PBC-specific AMA in serum from the child and his mother (see below).

**Figure 1:**
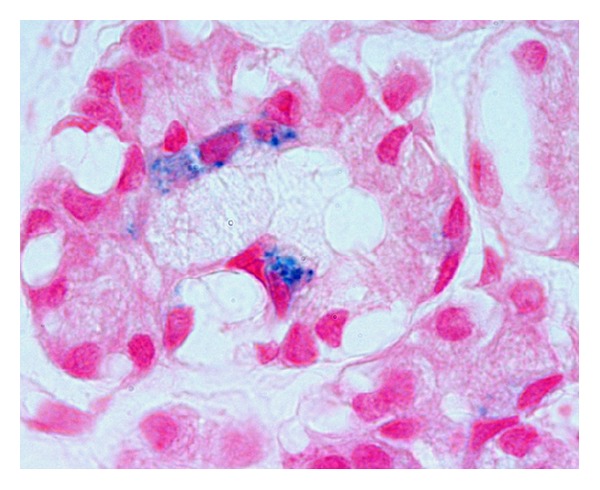
Granules of stainable iron (haemosiderin) within cytoplasm of epithelium of minor glands of the mucosa of the lower lip. Perls' technique with nuclear fast red counterstain, original magnification 1,000x.

The diagnosis of NH was confirmed at 12 days of age by microscopy of a lower lip mucosa biopsy specimen, which found haemosiderin granules within submucosal acinar gland cells (Figure 1). He was treated with intravenous desferrioxamine, sodium selenite pentahydrate, and N-acetylcysteine and was supported with blood and blood-product transfusions. At age 10 and 16 days he was given intravenous immunoglobulin (0.5 g/kg), and at 21 days he underwent single-volume exchange transfusion [22].

**Figure 2:**
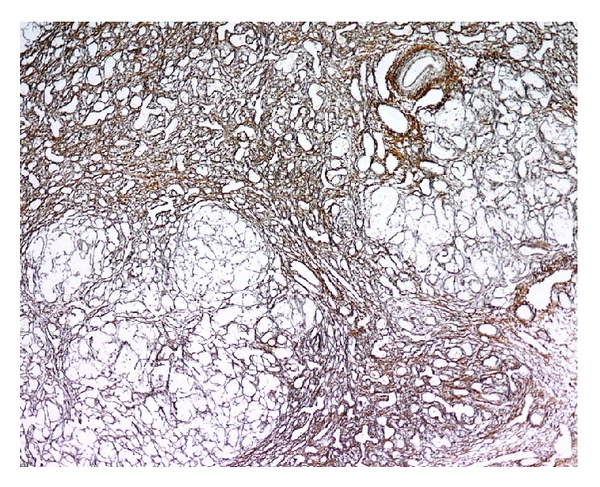
Hepatectomy specimen, postnatal age 30 days. Nodularity is apparent. The relatively stroma-poor nodules consisted of hepatocellular parenchyma, sometimes cholestatic. The intervening regions that exhibit more compact connective-tissue elements were sites of hepatocellular loss and stromal collapse, with neocholangiolar transformation of remaining parenchyma. Reticulin, original magnification 40x.

Poor clinical status prompted listing for liver transplantation (LT) at age 12 days. He received a cytomegalovirus mismatched (donor+/recipient-) split liver allograft at age 30 days. Examination of the explanted liver found postnecrotic cirrhosis with canalicular and hepatocellular cholestasis (Figure 2).

He made a slow but steady recovery. Following an isolated episode of lip smacking, and in view of microcephaly he underwent full neurological assessment, which found no cause for seizures; magnetic resonance imaging demonstrated normal brain structures with turricephaly. He was discharged home aged 92 days, 2 months after LT. Hip dislocation was successfully treated at age 10 months with reduction and casting. Allergy to milk, wheat, and egg antigens was diagnosed at age 13 months during evaluation of an urticarial rash. Epstein-Barr virus (EBV) infection was diagnosed at age 16 months (EBV DNA 136,907 copies/mL, blood) and at age 19 months upper respiratory tract infection accompanied fever and widespread lymphoadenopathy (EBV DNA 3,653,748 copies/mL). Adjustment in immunosuppressive regimen was rapidly followed by clinical improvement. Development was normal at age 22 months, without clinical-biochemistry evidence of hepatobiliary disease, although EBV viraemia persisted (EBV DNA 2,169,653 copies/mL). 

### 2.2. Autoantibody Testing

Serum samples obtained from the child at presentation and over the 17 months thereafter (including samples before and after LT) and from the mother at diagnosis of NH in the infant and 4 and 10 months after giving birth were tested for autoantibodies. An informed consent was obtained. Autoantibody testing complied with the principles laid down in the Declaration of Helsinki. Samples were stored at −80°C until use.

Autoantibodies were sought by indirect immunofluorescence (IFL) using as substrate rat liver/kidney/stomach tissue (KSL Slide, INOVA, San Diego, CA) and human epithelial (HEp-2) cells (INOVA) at a starting serum dilution of 1/20 to extinction. Autoantibodies to liver and nuclear antigens were tested by a combination of immunoassays including ELISAs (INOVA; EUROIMMUN, Lübeck, Germany), multiparametric line immunoassay (EUROIMMUN), and Western blot. These assays were based on purified M2 (AMA) antigen from porcine heart mitochondria; on individual human recombinant E2 pyruvate dehydrogenase complex (PDC), branched chain oxoacid dehydrogenase complex (BCOADC), and oxoglutarate dehydrogenase complex (OGDC) expressed in a baculovirus system (DIARECT, Freiburg, Germany); or on extracts from primate liver or from HEp-2 cell nuclei, as described [23–27].

AMA was initially detected by IFL. It was then characterised by line immunoassay and by an MIT3 ELISA whose target is a hybrid molecule comprising the major epitopes of PDC-E2, BCOADC-E2, and OGDC-E2 combined with native M2 antigen (M2-3E ELISA, EUROIMMUN). To investigate the specificity of the AMA response, reactivity against PBC-specific mitochondrial autoantigens was tested by in house ELISAs. These used recombinant PDC-E2, BCOADC-E2, and OGDC-E2 antigens (DIARECT) as individual targets. Briefly, wells of a flat-bottom plate (MaxiSorp, NUNC, Thermo Scientific, Roskilde, Denmark) were coated in triplicate with individual PDC-E2 (1 *μ*g/mL), BCOADC-E2 (1 *μ*g/mL), and OGDC-E2 (2 *μ*g/mL) diluted in phosphate buffered saline (PBS, Sigma-Aldrich, Coylton, Ayrshire, UK) containing 0.1% bovine serum albumin (BSA) at 37°C for 1 h. After a blocking step with 2% BSA for 1 h followed by 3 washes, serum samples (1/100) in 0.1% PBS/Tween20 (Sigma-Aldrich) were added to the coated wells and the plates were incubated for 1.5 h at room temperature on a shaker. After washing (3x), a horseradish peroxidase-conjugated anti-human IgG (INOVA) was added. The plates were held at room temperature for 45 minutes and the chromogenic substrate tetramethylbenzidine (INOVA) was added. The plates were then kept at room temperature for 15 min in the dark. Reaction was terminated with 100 *μ*L/well of 4N H_2_SO_4_ and absorbance (optical density, OD) at 450 nm was read against blank wells in a microplate reader (MRX, Dynex Technologies). 

IgG subclass-specific anti-PDC-E2 reactivity was investigated by in-house ELISAs, using horseradish peroxidase-labelled anti-IgG1, IgG2, IgG3, and IgG4 (SouthernBiotech, Birmingham, AL), as described [28]. Each sample was tested in triplicate and the mean optical density was calculated. 

## 3. Results

At time of presentation to our unit on day 7 of life, the infant's serum contained AMA of the IgG isotype at a titre of 1 : 80 by IFL; the mother's serum obtained at the same time contained AMA at a titre of 1 : 320 for IgG and 1 : 40 for IgM. Other autoantibodies, including anti-nuclear, anti-smooth muscle, anti-liver kidney microsomal, anti-double stranded DNA, and anti-extractable nuclear antigens, could not be demonstrated in either.

**Figure 3:**
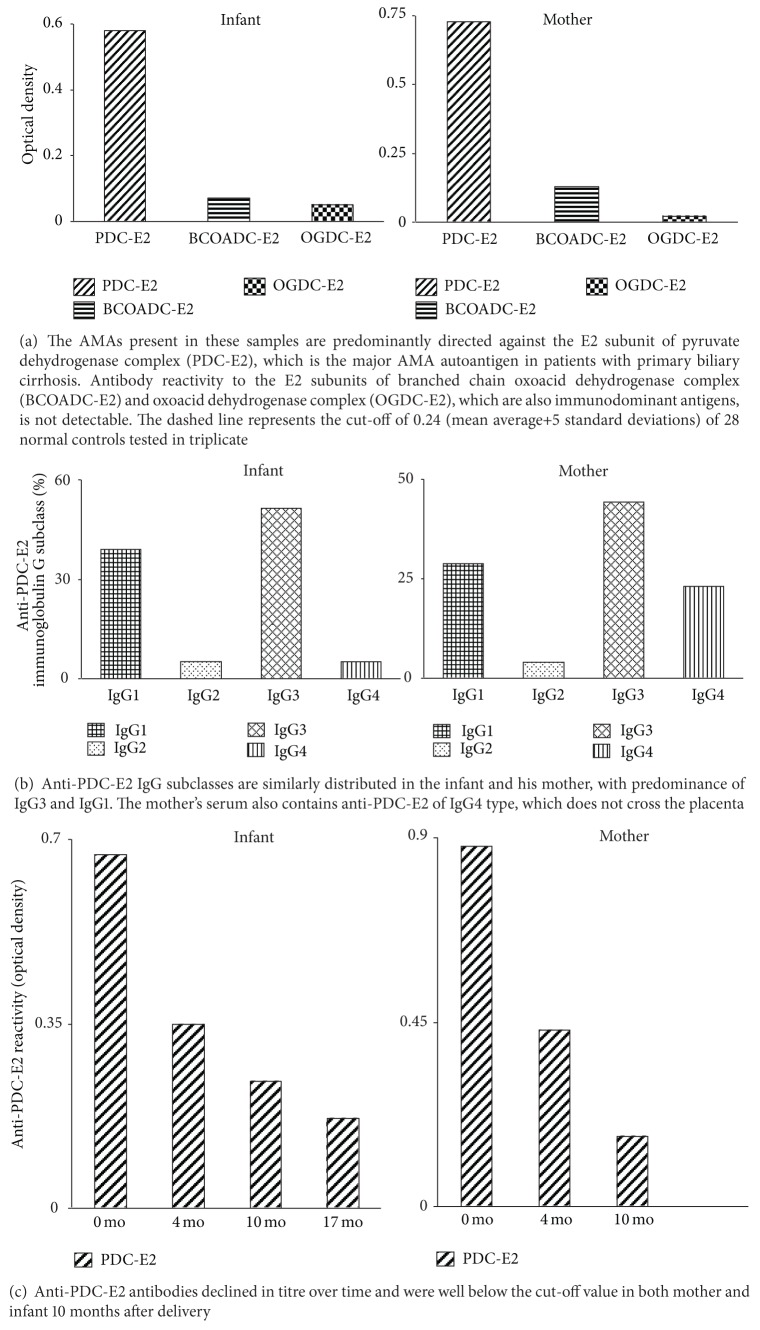
Antigen specific anti-mitochondrial antibody (AMA) reactivity in the infant and his mother tested by ELISA. Optical density (*y*-axis) and time elapsed since birth of child (*x*-axis) are indicated.

The AMA of infant and mother reacted only with PDC-E2 (Figure 3(a)) and belonged to the IgG class. The infant autoantibody belonged predominantly to the IgG3 and to a lesser extent to the IgG1 isotype (Figure 3(b)). There was no anti-PDC-E2 reactivity of the IgM or IgA isotype. A similar anti-PDC-E2 antibody distribution was seen in the mother, with the difference that IgG4 and IgM (1 : 40) anti-PDC-E2 antibodies also were present (Figure 3(b)). Concentrations of IgG2 anti-PDC-E2 antibodies were negligible in both mother and infant. At age 4 months (3 months after LT), the child's serum still reacted against PDC-E2, although the antibody titre was lower than that at diagnosis (Figure 3(c)). Anti-PDC-E2 reactivity also declined in the mother's serum (Figure 3(c)). Anti-PDC-E2 antibodies were borderline demonstrable in the child at age 10 months and were undetectable in contemporaneously obtained serum from his mother. At age 17 months, anti-PDC-E2 reactivity in the child had declined below the threshold of detectability (OD: 0.2; cut off: 0.24; Figure 3(c)). IgM- or IgA-class anti-PDC-E2 antibodies were never observed in the child.

## 4. Discussion

AMA, a liver-disease-specific autoantibody, is reported here for the first time in an infant with NH. All the evidence indicates that his AMA was of maternal origin, since it belonged to the placenta-crossing IgG class and showed a similar IgG3 and IgG1 pattern in mother and baby [29, 30]. The mother's serum also contained small quantities of AMA of IgG4 and IgM isotypes, which do not cross the placenta [29]. Both in mother and baby, AMA targeted PDC-E2, the major AMA antigen in PBC, but was peculiarly unreactive with BCOADC-E2 and OGDC-E2, which in PBC are usually recognised by sera that contain anti-PDC-E2 antibodies [20, 21, 31]. 

Whitington and colleagues described *in vitro* binding to hepatocytes by maternal antibodies of the IgG1 and IgG3 isotypes, thought to target liver alloantigens in infants with NH after crossing the placenta [8, 9]. IgGs isolated from these infants are able to bind to murine hepatocytes and to induce their necrosis through complement activation [8, 9], an event immunohistochemically reflected in the assembly of the terminal complement components on the hepatocytes of NH infants [10].

The AMA present in our mother-baby pair targets PDC-E2, an autoantigen typically recognised by the AMA of PBC patients, and belongs to the complement-fixing IgG3 and IgG1 isotypes, akin to the antibodies described by Whitington [8]. IgG3 and IgG1 antibodies are particularly efficient at activating the complement system and at recruiting cells bearing the Fc receptors that are capable of phagocytosis and antibody-dependent cell-mediated cytotoxicity [32–34]. Of note is that the ability of AMA to activate the complement system has been described in patients with PBC [35, 36].

If the IgG3/IgG1 AMA present in our infant recognised a pathogenetically relevant liver target, as proposed for PBC [37, 38], it may have contributed to the development of severe liver damage and failure. 

AMA has been occasionally described in liver disease in paediatric patients. Gregorio et al. reported a girl who at age 12 years developed autoimmune hepatitis with PBC-specific AMA [39]. AMA persisted throughout the course of her disease despite treatment; the patient died at age 24 years with liver failure, following several episodes of nonadherence to treatment and spontaneous bacterial peritonitis [39]. More pertinent to our case, placentally transferred PDC-E2-targeting IgG3 and IgG1 AMAs have been reported in 2 infants with acute liver injury that lasted until the antibody disappeared [28]. 

The AMA present in the mother of our patient with NH is of interest for two reasons: it partially belonged to the IgM isotype, suggesting that it was produced as part of a primary autoimmune response; it declined in concentration over time and eventually disappeared, implying that the triggering autoantigenic stimulus had subsided. A loss of AMA, though occasionally reported, is unusual, since AMA tends to persist once detected, heralding the development of PBC [40]. The mother of the infant reported here is regularly monitored for a possible reappearance of AMA, especially in the case of a new pregnancy.

Recent reports show that AMA can be detected in some 40% of patients with acute liver failure when the antibody is sought with a highly sensitive MIT3 ELISA [41, 42]. In our study an MIT3 ELISA was used to confirm IFL results; it demonstrated AMA in both the infant and his mother. Akin to the AMA characterised in the present study, the AMAs described by Leung et al. using MIT3 ELISA were transient [41]. Their disappearance was attributed to lack of genetic susceptibility to PBC. The real prevalence and possible involvement of AMA in the causation of the severe liver damage accompanying NH will need to be investigated using similarly sensitive techniques in a series of affected infants.

Although we cannot definitively link AMA with liver injury in this infant, and NH is not a known complication of pregnancy in women with PBC, no other cause of liver injury was identified. We also cannot rule out AMA as being associated with the liver disease seen in this infant, as the transplacental passage of AMA has been reported in infants with liver injury of unknown cause [28]. As well, the prevalence of AMA in NH patients and/or their mothers has not been evaluated. 

## List of Abbreviations

AMA: Antimitochondrial antibodies
ALT: Alanine transaminase
AST: Aspartate aminotransferase
BCOADC-E2: Branched-chain oxoacid dehydrogenase complex E2 subunit
EBV: Epstein-Barr virus
ELISA: Enzyme linked immunosorbent assay
IgG/A/M: Immunoglobulin G/A/M
IFL: Indirect immunofluorescence
INR: International Normalised Ratio
LT: Liver transplantation
NH: Neonatal haemochromatosis
OGDC-E2: Oxoglutarate dehydrogenase complex E2 subunit
OD: Optical density
PBC: Primary biliary cirrhosis
PDC-E2: Pyruvate dehydrogenase complex E2 subunit
PBS: Phosphate buffered saline.
